# Orthoptic Services in the UK and Ireland During the COVID-19 Pandemic

**DOI:** 10.22599/bioj.153

**Published:** 2020-06-05

**Authors:** Fiona Rowe, Lauren Hepworth, Claire Howard, Steven Lane

**Affiliations:** 1 University of Liverpool, GB; 2 Salford Royal NHS Foundation Trust, GB

**Keywords:** Orthoptist, COVID-19, Coronavirus, Service delivery, Survey

## Abstract

**Aim:**

COVID-19 has widely impacted hospital services. The purpose of this study was to determine the impact of COVID-19 on Orthoptists and their clinical practice in the UK, Ireland, and Channel Islands.

**Methods:**

We conducted a prospective survey-based cross-sectional study using an online survey aiming for coverage of orthoptic departments across the UK, Ireland, and Channel Islands. We circulated the online survey through the British and Irish Orthoptic Society that reaches over 95% of UK and Irish orthoptic services, and through social media and orthoptic research networks.

**Results:**

The survey response rate was 79%. The survey was completed by orthoptic departments, on average 10 days post lockdown. Many orthoptic services were cancelled/paused with remaining services largely reserved for emergency cases and urgent care. A substantial rise in tele-consultations was reported by 94%, which largely consisted of telephone and video calls and which was regarded generally as working well. Barriers to tele-consultations were mainly IT related but with concerns also raised regarding ethical and confidentiality issues. Shortage of personal protective equipment (PPE) was reported by one third of departments along with issues relating to conflicting information about the use of PPE.

**Conclusions:**

We have reported information on the changing face of orthoptic clinical practice during the COVID-19 pandemic. The survey has highlighted emerging tele-consultation practice and the importance of centralised profession-specific guidelines.

## Introduction

**Figure 1:**
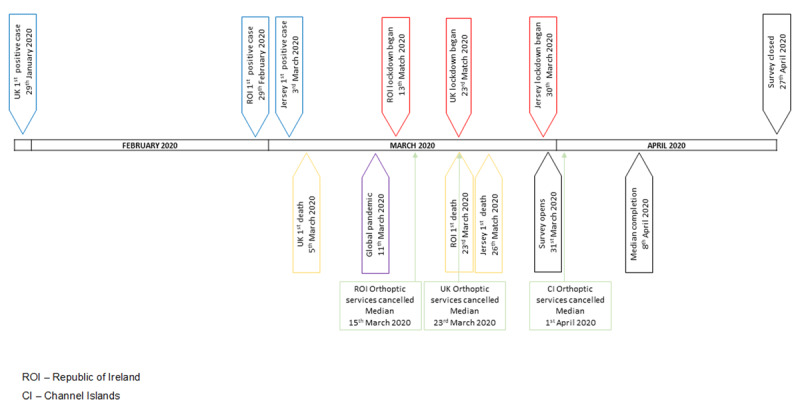
Key dates during COVID-19.

COVID-19 (Corona Virus Disease 2019) is caused by coronavirus and is also known as SARS-CoV-2, 2019 Novel Coronavirus, and nCoV. The virus was first reported in Wuhan, China in late 2019 (Zhu et al. 2020). The first cases testing positive in the UK (including England, Scotland, Wales, and Northern Ireland) were on 29 January 2020, in Ireland on 29 February, and Jersey on 3 March with the first deaths on 5, 23, and 26 March, respectively (Dept. Health, Ireland 2020; Dept. Health & Social Care, UK 2020a; Office of Superintendent Registrar, Jersey 2020). The World Health Authority declared a pandemic on 11 March (Nussbaumer-Streit et al. 2020). Key dates relating to COVID-19 are outlined in Figure 1.

Shortly afterwards, on 16 March, the UK government advised the public to work at home where possible and avoid social gatherings. On 20 March, UK schools, pubs, restaurants, and social venues were closed with recommendations to stay at home, unless for essential movement. ‘Stay at home’ lockdowns began on 12 March in Ireland, 23 March in the UK, and 30 March in Jersey. With increased admissions to hospitals and alongside government recommendations for social distancing, substantial changes to clinical practice were required. Social distancing at this time was defined as remaining at least two metres away from people who are not members of your household. Planned changes to clinical practice were under discussion in February as cases began to rise in the UK and in light of rapidly escalating circumstances in European Union (EU) countries (e.g. Italy, Spain, and France) (Dept. Health & Social Care, UK 2020b).

Attention naturally focussed on Emergency Departments and intensive care units. Other areas of focus included the documentation of rates of virus spread and morbidity, vaccine and therapeutic developments, alongside societal impact through enforced restrictions and personal isolation. However, the changes within routine clinical care within non-critical care settings have received less attention. Yet, within such care settings, clinical practice has changed with remarkable speed. One setting is that of orthoptic services. Orthoptists are autonomous allied health professionals who assess, diagnose, treat, and monitor a variety of eye disorders (BIOS 2020a). Most Orthoptists in the UK are employed by the NHS and work in:

- hospitals (inpatient wards, outpatients, and theatre)
- community clinics
- rehabilitation centres
- special schools and child development centres
- mainstream schools.

The purpose of this study was to determine the impact of COVID-19 on Orthoptists and their clinical practice.

## Methods

A prospective cross-sectional survey was undertaken across orthoptic departments registered with the British and Irish Orthoptic Society (BIOS), covering the UK, Republic of Ireland, and Channel Islands. The survey was approved by the University of Liverpool Research Ethics Committee (Ref. 7637). Recorded informed consent was sought. The first page of the survey contained standard information which would be found in a participant information sheet, followed by four statements which acted as a consent form.

Initial development of survey questions took place with collaboration between the University of Liverpool and clinical orthoptic colleagues working in hospitals within the North West of England. Independent, non-vision peer review was sought for the survey content within the Institute for Population Health at the University of Liverpool from colleagues already involved in national COVID-19 research activity. Questions addressed the impact to orthoptists personally in their working lives, changes to their working environment, changes to their working practice and how they consult with patients, and access by orthoptists to professional support and guidelines (Supplementary Table 1).

**Table 1:** Orthoptic department staffing numbers.

Staff levels	Median	Range

Number of orthoptists	7	1–32
FTE orthoptists	6	1–30
Number of orthoptic assistants	0	0–9
FTE orthoptic assistants	0	0–9
Number of orthoptic administrators	0	0–9
FTE orthoptic administrators	0	0–6

The survey was administered using Qualtrics software (Qualtrics USA 2019) and was circulated through BIOS with emails of the survey link to all registered heads/leads of orthoptic services (total of 175 organisations, e.g. Trusts and Health Boards). The survey opened on 31 March 2020 and closed on 27 April 2020, a four-week duration to allow for Easter holidays and increased clinical and administrative workload due to COVID-19. During the first week, updates on the survey completion rate were posted through the BIOS Leads of Orthoptic Practice (LOOP) online forum on a one-to-two-day basis. After 10 days, further email requests were sent to heads/leads as a reminder for survey completion. Whilst the survey remained open, frequent updates on survey completion rates were posted using social media platforms, e.g. Twitter.

Our aim was to capture a response from every orthoptic department in the UK, Republic of Ireland, and Channel Islands where possible. However, an acceptance rate of 30% initial response to this survey was determined as appropriate given the current climate.

Analysis of survey results were primarily descriptive. Survey results were imported from Qualtrics software. SPSS software (IBM, version 25) was used for descriptive analysis of the data. Open text survey responses were uploaded into NVivo software (QSR International) (QSR Int. 2018) for qualitative compilation of orthoptic comments. Open text responses were coded by sentence. A thematic approach to analysis of this qualitative data was adopted. Two researchers independently coded the first five survey responses using line-by-line coding resulting in two preliminary coding lists. Each open-text question was dealt with separately. The same two researchers compared the codes applied. Codes were grouped for similar content and a narrative summary produced for each survey question that generated open-text responses.

## Results

### Survey responses

Responses were received from 163 orthoptists providing data for 138 departments (25 duplicates) with a department response rate of 79%. Duplicate responses from the same department were combined to capture a single response from each department. Each department was asked to specify if they linked to orthoptic departments in other hospitals covered by the same Trust. Free text response boxes allowed for specification of different situations across jointly managed departments.

**Figure 2:**
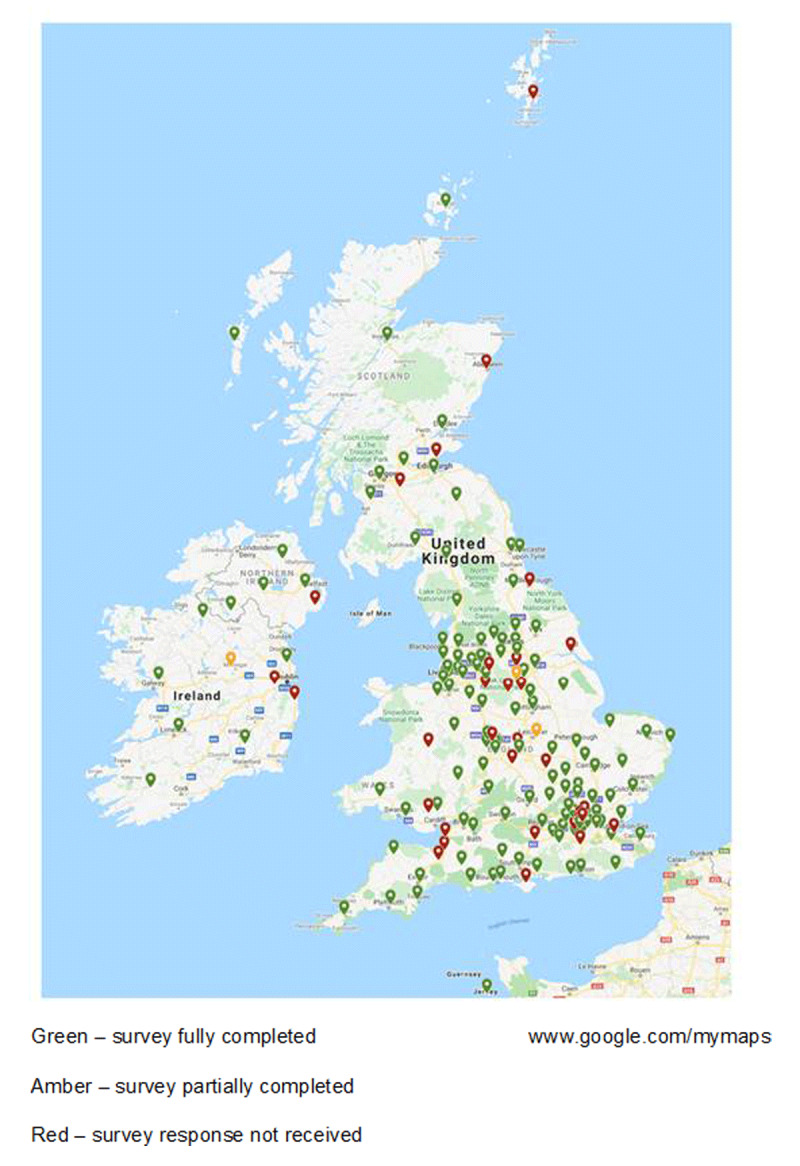
Survey completion map by department.

Responses were received from across the UK (n = 130), Republic of Ireland (n = 7), and Channel Islands (n = 1) (Figure 2). The average completion date for the survey was 10 April 2020 (median 8 April) ranging from 31 March to 27 April for survey completion dates. The mean was 10 days (SD 7.5) from survey release to completion and 19 days (SD 8.6) from the respective lockdown dates.

Department staffing is outlined in Table 1. The populations served by the hospitals represented in this survey ranged from 100,000 to 7,000,000 (median 450,000).

### Patient consultations

**Figure 3:**
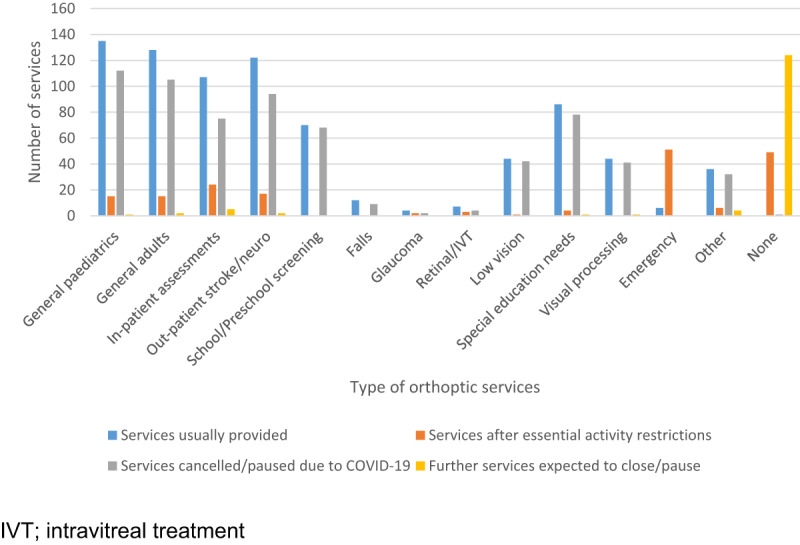
Provision of clinical services pre- and during COVID-19. Other services inclusive of chalzion, ocular toxicity, contact lens, refraction, theatre, visual stress, functional visual assessment, biometry/cataract, imaging, optical coherence tomography (OCT), cysts, oncology, YAG laser, neuro-ophthalmology, thyroid, botulinum toxin and visual field clinics.

The clinical services normally provided pre-COVID-19 are outlined in Figure 3 along with the clinical services provided following UK Government recommendations on avoiding non-essential activity which were issued on 16 March 2020. Intention to lockdown was given on 20 March 2020 and subsequent lockdown occurred on 23 March 2020 in the UK (12 March in Ireland and 30 March in Jersey). The average date (with lockdown) of cancelled/paused services due to COVID-19 was 21 March 2020 (median 23 March) in the UK, 15 March 2020 (median 16 March) in Ireland, and 1 April 2020 in the Channel Islands. These dates are displayed in Figure 1.

Method of consultation was reported as face-to-face (n = 92), telephone consultations (n = 129), video consultations (n = 12), and other (n = 41). Other methods of consultation were inclusive of: no consultations if working at home, provision of only urgent/emergency face-to-face appointments, text messages, and letters. Some departments stated that these consultations were backed-up with a letter to summarise the discussion.

**Figure 4:**
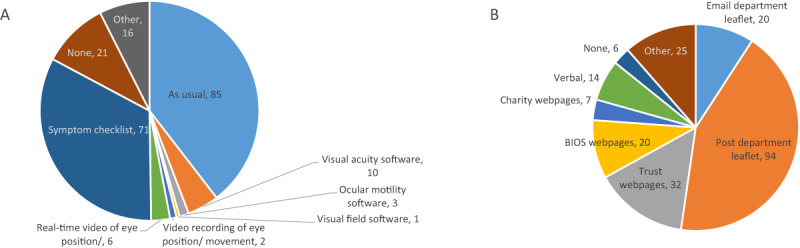
COVID-19 consultation options. **A** – how orthoptists reported gathering visual information during consultations Other; red reflex photos, proforma, Royal College of Ophthalmology, BIOS guidelines, risk stratification tool. **B** – what resources orthoptists reported providing to their patients during COVID-19 Visual acuity software (iSight app; DigVis app). Other; post patches for amblyopia, post patch/prism for stable diplopia, use of iSight app.

Methods of how information was gathered during consultation are outlined in Figure 4. When asked about provision of usual care, one department stated they were continuing to provide usual care, 28 were providing partial usual care (22 at an estimated <25% of usual care), two were unsure, and 105 were not providing usual care. Dates relating to the start of changed services are shown in Table 2.

**Table 2:** Time points for onset of changed services.

	Overall (n = 138)			

	Median		Range	

Outpatients cancelled	21 March 2020			6 March–1 April
Inpatients cancelled	23 March 2020			6 March–8 April
Tele-consultation start	23 March 2020			10 March–2 April
Work at home start	25 March 2020			16 March–23 April
	**UK (n = 131)**		**Ireland (n = 7)**	

	**Median**	**Range**	**Median**	**Range**

Outpatients cancelled	23 March 2020	10 March–30 March*	15 March 2020	6 March–23 March
Inpatients cancelled	23 March 2020	12 March–8 April*	15 March 2020	6 March–28 March
Tele consultation start	23 March 2020	10 March–2 April	19 March 2020	16 March–23 March
Work at home start	25 March 2020	16 March–23 April	23 March 2020	25 March–27 March

* *Note*: Channel Islands cancelled services on 1 April 2020.

In response to the question ‘how are you advising patients on new and on-going treatment’, one treatment that was consistently stopped by departments was use of atropine for amblyopia, with some specifying a move to conventional patch occlusion. Different approaches were taken with regard to conventional patch occlusion, with some continuing as previously prescribed, others reducing the treatment dose depending on original dose level, setting maintenance occlusion, or stopping all occlusion until the patient could next be seen. In the case of adult reviews, symptoms were the focus of aiding treatment decisions, with one consistent new treatment being occlusion for diplopia. There was little mention of prism provision due to lack of face-to-face consultations.

**Figure 5:**
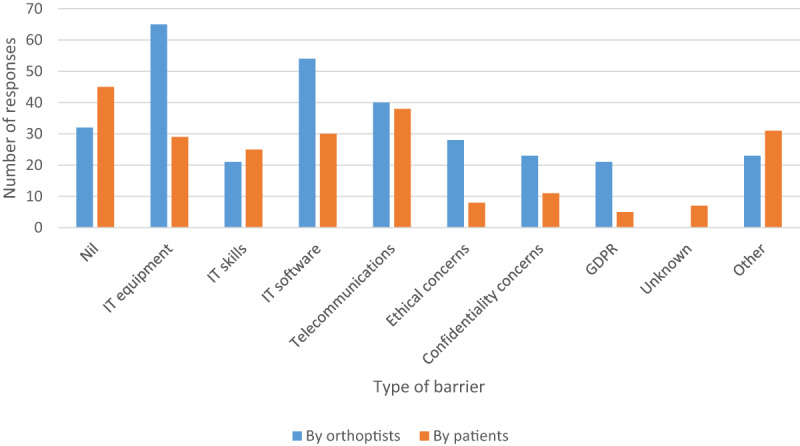
Barriers experienced with alternative consultations during COVID-19 by orthoptists and patients/parents. Other; patients unwilling to come to hospital because of COVID-19 risk, language barriers, lack of interpretors, incorrect telephone contact numbers on records, patients not answering phone, poor hearing, age, IT literacy, no mobile or computer access.

Barriers to providing alternative consultations are outlined in Figure 5 for those experienced personally by the orthoptists, and for those considered by the orthoptists as being barriers for patients. One response stated that ‘technology could not replace in-person consultations’. For those requiring extra support, the recurring requirement was provision of IT hardware and software to allow home working. Even those able to work from home reported that ‘improvements in IT could allow more efficient working’. Some departments reported concern in relation to the ‘lowering of clinical standards’, and delays in diagnosis and starting treatment due to the lack of face-to-face appointments or ability to assess vision remotely. Specific issues reported in relation to this were the ‘inability to initiate or change occlusion regimes safely’, or assess ‘required prism strength, thus having to resort to occlusion for diplopia’.

When asked about practice changes that were working well, orthoptists reported that ‘patient contacts have been largely positive’. Telephone consultations had ‘prevented unnecessary appointments, for example, when a child had not worn their glasses or occlusion’. Patients and families also reported that these telephone contacts were well received. The few departments using video consultations reported these to be working well with a desire from other departments to move to video consultations. Communication amongst teams was reported as largely positive, with the ‘instigation of daily catch ups’ in many departments. Good ‘teamwork and team spirit’ were commonly reported. Other positives include being able to complete activities for which previously it was difficult to find sufficient time, e.g. continuing professional development, audits, and mandatory training.

### Personal working issues

**Figure 6:**
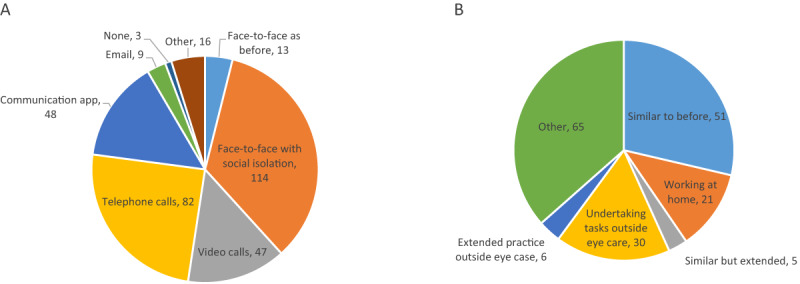
Work practices during COVID-19. **A** – how orthoptists report working with colleagues during COVID-19. Other; social distancing not working, all staff wearing face masks. **B** – the work situation reported by orthoptists during COVID-19. Other; mixed work, skeleton staff for urgent cases, cut-back services, tele-consultations, contingency planning, redeployment, triage service, audit, business planning, moved location.

Current lockdown working practices are outlined in Figure 6. Only 21 reported working from home when asked about their current working situation, whereas 84 departments reported a date for when working from home started.

Issues faced by orthoptists at work included travel restrictions (n = 16), transport issues (n = 21), lacking training (n = 23) and lack of information (n = 25), conflicting information (n = 61), lack of personal protective equipment (PPE; n = 34), redeployment (n = 64), childcare issues (n = 4), and other issues (n = 31; additional COVID-19 training, self-isolation, not allowed to work from home, anxiety).

When asked what redeployment roles have taken place or are planned, a high proportion of departments reported that ‘whilst redeployment was planned it was still unclear what roles may be taken on and when redeployment may happen’. The majority reported the specific areas they had already been redeployed to or were expected. The most mentioned roles included ward care assistant, administration, including call centres, and other roles within the ophthalmology department (e.g. eye casualty). Others were contributing to the daily working of the hospital (e.g. portering and housekeeping) or directly to COVID-19 (swab testing, doffing and donning, fit mask testing/training). Not all redeployment roles were within the hospital setting, with some aiding community nurses with eye care.

Responses specific to PPE included shortage (n = 33), use per Trust guidance (n = 87), use per BIOS guidelines (n = 28), use per Public Health England (PHE) guidance approved by all four UK nations (n = 42), and other (n = 41; conflicting information regarding PPE use, use of Perspex screens, lack of hands-on training, department slow to implement PPE, told of low risk, no PPE supplied at all). The PHE guidance regarding PPE changed on 2 April 2020 to state disposable gloves, aprons, and eye protection should be worn if needed for direct patient contact, which the recommendation by the Royal College of Ophthalmology guidelines for eye outpatients linked from the BIOS website (Public Health England, 2020; Royal College of Ophthalmology, 2020b).

The response to access to guidelines for COVID-19 in relation to their role included guidelines from local trust/health board (n = 128), government bodies (n = 91), BIOS (n = 86), Royal College of Ophthalmology (n = 64), and HCPC (n = 48).

The survey also asked if it was considered more difficult to focus on work during the COVID-19 lockdown. Thirteen strongly agreed that focus on work was difficult, 51 agreed, 9 were neutral, 10 disagreed, and 2 strongly disagreed.

## Discussion

Our survey achieved a high response rate of 79% within four weeks of first opening for completion by orthoptic departments in the UK, Ireland, and the Channel Islands. This allowed a rapid collation of information about the impact of orthoptic care during the initial changes within health services. Key dates for government recommendations were 12 March (Ireland), 23 March (UK) and 30 March (Jersey) (gov.uk 2020a, 2020b). The survey was completed, on average, within 19 days of these dates.

Pre-COVID-19, a wide range of orthoptic services were provided with considerable numbers of these services cancelled/paused due to COVID-19. Services remaining during essential-activity restrictions included general paediatric/adult care, in-patient assessments, outpatient stroke/neuro care, glaucoma, retinal/intravitreal treatment (IVT), special education needs, and emergency care. About 50 departments stated no services were being provided; often community eye care and school screening services. These changes to orthoptic services mirror many of the wide range of clinics offered by ophthalmology departments (e.g. paediatric, neuro, cataract, etc.) and other hospital services (orthopaedics) in continuing to see emergency cases, an increase in telemedicine and reduction in face-to-face assessments (Korobelnik et al. 2020; Lai et al. 2020; Nagra et al. 2020; Nair et al, 2020; Shabto et al. 2020; Thaler et al. 2020). Many services continued as remote consultations (telephone/video calls, letters) whilst face-to-face consultations in clinic were primarily for emergency cases and urgent care. In other ophthalmic and optometric settings, telephone triage has been used to establish at-risk and emergency patients (Nagra et al. 2020). One survey of ophthalmologists in India reported three quarters providing tele-consultations (Nair et al. 2020) in comparison to 93% reported in our survey.

Orthoptists continued to gather clinical information in usual ways, but some were using software to gather information about visual function (e.g. use of iSight app for visual acuity and use of photos/video recordings for ocular alignment and motility). A symptom checklist was used by 51%. Methods of gathering information raises issues regarding access to apps that are validated for clinical use, but also used for self-administration by parents/patients, with the latter posing questions about the reliability and validity of apps when self-administered. One ophthalmic study reported the absence of a comprehensive evidence base for telemedicine in ophthalmology and confirmed that research into self-administered visual acuity measurements is limited with use of apps and smartphone imaging by clinicians, but not by patients (Nagra et al. 2020). Treatment options changed with atropine occlusion for amblyopia therapy being stopped in line with guidance from the Royal College of Ophthalmology (RCOphth 2020a). Conventional occlusion continued but often at altered ‘maintenance’ doses. Occlusion also became the primary treatment for acute onset diplopia.

Although travel restrictions and transport issues were problematic for some, the main concern raised by orthoptists regarded information and training directed at COVID-19. This particularly concerned conflicting information plus redeployment and PPE anxieties. Most orthoptists continued to work face-to-face with clinical colleagues although with social isolation practices in place where possible. Few (15%) worked solely from home, with most working as usual in eye clinics whilst others were redeployed fully or partially outside eye care. One quarter of departments reported PPE shortage. However, when provided, this was used according to Trust, NHS, and/or professional guidelines. As reported throughout the pandemic, PPE access and use was a key issue and most orthoptists cited problems with PPE such as conflicting information on its use plus shortages. Use of PPE is important in eye care (Pult et al. 2020) as ophthalmic practice carries unique risks (Houghton et al. 2020). Use of protective shields on ophthalmic equipment is required along with appropriate infection control training for staff (Lam et al. 2020). Clear communication of PPE guidelines with appropriate training and sufficient provision of PPE is crucial (Lam et al. 2020).

Common barriers reported by orthoptists related primarily to IT issues and telecommunications. However, concerns were also raised regarding ethical and confidentiality issues when using remote consultation options. A review of telemedicine in ophthalmic practice highlighted the importance of appropriate design and implementation of telemedicine for eye care services to avoid such barriers (Saleem et al. 2020). Importantly, the Health and Care Professions Council (HCPC – regulatory body for orthoptists in the UK) issued a statement: ‘In highly challenging circumstances, professionals may need to depart from established procedures in order to care for patients… Our regulatory standards are designed to be flexible’ (Health and Care Professions Council, 2020). This is arguably of some reassurance when working in such different ways under changing circumstances. Nair and colleagues also cited the importance for regulatory bodies to issue appropriate guidance (Nair et al. 2020).

An important contribution was the BIOS response to COVID-19 early on with a section of the professional website assigned specifically to COVID-19 information made available from 18 March 2020 (BIOS 2020b) and covering general advice, guidance of orthoptists, workplace and employment, returning to the NHS, students, and temporary membership. General advice covered links to the latest government advice for England, Wales, Scotland, Northern Ireland, and the Republic of Ireland. Guidance for orthoptists covered aspects related to continued work within the NHS, patient contact, use of PPE, tele-consultation guidance, risk for vulnerable staff, insurance, training, and patient resources. Workplace guidance covered self-isolation, pregnancy, and insurance. BIOS offered temporary registration and membership during this time. Further, they offered specific COVID-19 online discussion forms for members to share advice and receive updates. Whilst condition-specific recommendations have been provided for some ophthalmic conditions such as glaucoma and retinal IVT services (Korobelnik et al. 2020; Shabto et al. 2020), BIOS provided one site for profession-specific information.

We acknowledge that these survey results are a snapshot of orthoptic services over a four-week period in April 2020 and that response to the pandemic at local and national levels are changeable and ongoing. Next steps are to consider planning for the recovery phase within orthoptic services. BIOS provides information for this. There are opportunities to take positive working practices established during the pandemic and embed these into future practice. Questions are raised for future research, such as exploring the validity of app testing remotely in self-administration mode by parents/patients. A follow-up survey is planned in the future to capture how orthoptic services and practice have changed since this initial response.

## Conclusions

The COVID-19 pandemic forced a complete change in orthoptic practice with face-to-face clinical consultations mainly preserved for urgent care. Clinical practice continued, but scaled down, through remote consultations with use of video and telephone calls. The latter raise the concept of using software apps to test visual function and facilitate such consultations. The primary barrier to remote working was IT issues and one main problem experienced by orthoptists was the lack of PPE and conflicting guidance on use of PPE. A single point of contact for provision of professional information is crucial and was facilitated by the BIOS professional organisation who, from a very early stage, provided access to highly relevant and timely information to support on-going orthoptic practice and changes to practice.

## Data Accessibility Statement

Data is available from the lead author on reasonable request.

## Additional File

The additional file for this article can be found as follows:

10.22599/bioj.153.s1 Supplemental Table 1. Survey questions.
