# Supplementary material for: Orthoptic Services in the UK and Ireland During the COVID-19 Pandemic
Source: Br Ir Orthopt J. 2020 Jun 5;16(1):29–37. doi: 10.22599/bioj.153 (PMC7510392; doi:10.22599/bioj.153)
Supplement: Supplemental Table 1. — Survey questions. [file bioj-16-1-153-s1.pdf]

Please indicate which Trust / hospital you work for?

This information is to check coverage of the survey across the UK and to collate information where there may be duplicate responses from more than one orthoptist from the same department.

Please list all the orthoptic departments, including those in other hospitals, that your service covers:

What is the approximate population catchment for your department – if known?

What is the staffing size of your department?

Please indicate the number of individuals and FTE for orthoptists, orthoptic assistances, orthoptic administrators, etc

	Number of individuals	FTE
Orthoptists		
Orthoptic assistants		
Orthoptic admin		

Patient consultation

What clinical services do you normally (pre-COVID19) provide for patients?

Please tick all that apply:

- ☐ General paediatrics
- ☐ General adults
- ☐ In-patient assessments (e.g. stroke/neuro)
- ☐ Out-patient stroke/neuro

- School screening
- Falls
- Glaucoma monitoring
- Age-related macular degeneration
- Low vision
- Special educational needs
- Visual processing difficulties
- Other – please specify:

Since Government recommendations on essential activity, what clinical services do you provide now for patients?

Please tick all that apply:

- None
- General paediatrics
- General adults
- In-patient assessments (e.g. stroke/neuro)
- Out-patient stroke/neuro
- School screening
- Falls
- Glaucoma monitoring
- Age-related macular degeneration
- Low vision
- Special educational needs
- Visual processing difficulties
- Other – please specify:

If services have been cancelled/paused due to COVID-19, on what date did this take place?

Which services have been cancelled/paused?

Please tick all that apply:

- ☐ None
- ☐ General paediatrics
- ☐ General adults
- ☐ In-patient assessments (e.g. stroke/neuro)
- ☐ Out-patient stroke/neuro
- ☐ School screening
- ☐ Falls
- ☐ Glaucoma monitoring
- ☐ Age-related macular degeneration
- ☐ Low vision
- ☐ Special educational needs
- ☐ Visual processing difficulties
- ☐ Other – please specify:

What clinical services do you expect to have cancelled in the near future?

Please tick all that apply:

- ☐ None
- ☐ General paediatrics
- ☐ General adults
- ☐ In-patient assessments (e.g. stroke/neuro)
- ☐ Out-patient stroke/neuro
- ☐ School screening
- ☐ Falls
- ☐ Glaucoma monitoring
- ☐ Age-related macular degeneration
- ☐ Low vision
- ☐ Special educational needs
- ☐ Visual processing difficulties

- ☐ Other – please specify:

How are you providing consultations currently with patients?

Please tick all that apply:

- ☐ Face-to-face clinic appointment
- ☐ Telephone call
- ☐ Video call
- ☐ Other – please specify:

How are you currently gathering visual information during consultations?

Please tick all that apply:

- ☐ Usual practice with face-to-face clinic appointment
- ☐ Software app for visual acuity – please specify:
- ☐ Software app for eye movements – please specify:
- ☐ Software app for visual fields – please specify:
- ☐ Video recording of eye position and movements
- ☐ Real-time assessment of eye position and movements during video call
- ☐ Symptom checklist
- ☐ Other – please specify: Proforma

How are you advising patients on new or on-going treatment?

e.g. when commencing new treatment, advising on on-going treatment – whether to continue on same regime or changing regime

How are you providing resources to patients?

Please tick all that apply:

- ☐ Department leaflets/factsheets sent by email
- ☐ Department leaflets/factsheets sent by post
- ☐ Department leaflets/factsheets available on Trust webpages
- ☐ Information leaflets/factsheets available on BIOS webpages
- ☐ Information leaflets/factsheets available on Charity webpages – please specify:
- ☐ Other – please specify:

Do you feel you are able to provide usual orthoptic care under the current COVID-19 revised working circumstances?

- ☐ Yes
- ☐ Partially
- ☐ No
- ☐ Unsure

If yes or partially, can you provide an estimate of how many orthoptic patients are still receiving usual care in your area?

Please tick:

- ☐ 100%
- ☐ 75-99%
- ☐ 50-74%
- ☐ 25-49%
- ☐ <25%

Comment:

Have you experienced any barriers to alternative ways of providing consultations - from your personal situation?

Please tick all that apply:

- ☐ No barriers experienced
- ☐ IT equipment
- ☐ IT skills
- ☐ Telecommunications
- ☐ IT software
- ☐ Concerns regarding ethics
- ☐ Concerns regarding confidentiality
- ☐ GDPR
- ☐ Other – please specify:

Have you experienced any barriers to alternative ways of providing consultations - because of the patient's situation?

Please tick all that apply:

- ☐ No barriers experienced
- ☐ IT equipment
- ☐ IT skills
- ☐ Telecommunications
- ☐ Access to appropriate software
- ☐ Concerns regarding ethics
- ☐ Concerns regarding confidentiality
- ☐ GDPR
- ☐ Other – please specify:

How are you working currently with colleagues?

Please tick all that apply:

- ☐ Continued face-to-face as before
- ☐ Continued face-to-face with social distancing
- ☐ Video conferencing
- ☐ Telephone calls
- ☐ Communication software app
- ☐ Other – please specify:

Personal working practice

What is your current working situation?

Please tick all that apply:

- ☐ Similar schedule and activities
- ☐ Mostly working from home
- ☐ Same activities as before, but extended schedule due to lack of personnel
- ☐ Similar schedule but doing different tasks outside of eye care
- ☐ Extended working hours, mostly outside of eye care.
- ☐ Other – please specify:

What strategies are in place to protect vulnerable orthoptic staff (those who require shielding due to health concerns?

e.g. orthoptic duties without patient contact, work from home, etc.

What COVID-19 issues are being faced by Orthoptists in your department?

Please tick all that apply:

- ☐ Travel restrictions
- ☐ Transport issues

- ☐ Lack of training
- ☐ Lack of information
- ☐ Conflicting information/guidelines
- ☐ Lack of PPE
- ☐ Redeployment
- ☐ Other – please specify:

What are the key COVID-19 issues for Orthoptists in relation to personal protective equipment (PPE)?

Please tick all that apply:

- ☐ Shortage of PPE
- ☐ Using appropriate PPE according to Trust guidelines
- ☐ Using appropriate PPE according to BIOS guidelines
- ☐ Using appropriate PPE according to PHE guidelines
- ☐ Other – please specify:

Where redeployment has taken place or is planned, what roles are Orthoptists taking on?

What Trust support has been provided to orthoptists?

e.g. access to counselling support, flexible hours, work from home

What additional Trust support is needed for orthoptists?

e.g. access to counselling support, flexible hours, work from home

What access do you have to guidelines for COVID19 in relation to your work?

e.g. Trust guidelines, national NHS guidelines, national professional society guidelines

Prioritisation

When did your service start to implement changes in response to COVID-19?

Please indicate approximate dates for when changes were made:

	Date of change:
☐ Out-patient appointments cancelled	
☐ In-patient assessments cancelled	
☐ Patient telephone consultations commenced	
☐ Work from home started	
☐ Other – please specify all:	

What Orthoptic services have been prioritised and why?

Experiences

In the time since changes to your practice have been made;

What seems to be working well?

What is not working well?

Is there a message you would like to share with the orthoptic community?

Is there an important piece of learning in recent weeks you would like to share with the orthoptic community?

How much do you agree with the following statement?

‘I have found it more difficult than usual to focus on work-related tasks’

- ☐ Strongly disagree
- ☐ Disagree
- ☐ Neutral
- ☐ Agree
- ☐ Strongly agree
